# Extracellular Vesicles: A Therapeutic Option for Liver Fibrosis

**DOI:** 10.3390/ijms21124255

**Published:** 2020-06-15

**Authors:** Stefania Bruno, Giulia Chiabotto, Giovanni Camussi

**Affiliations:** Department of Medical Sciences and Molecular Biotechnology Center, University of Torino, 10126 Torino, Italy; giulia.chiabotto@unito.it (G.C.); giovanni.camussi@unito.it (G.C.)

**Keywords:** exosomes, stem cells, collagen deposition, inflammation, acute liver injury, chronic liver damage

## Abstract

Extracellular vesicles (EVs) are a heterogeneous population of small membrane vesicles released by all types of cells in both physiological and pathological conditions. EVs shuttle different types of molecules and are able to modify the behavior of target cells by various mechanisms of action. In this review, we have summarized the papers present in the literature, to our acknowledge, that reported the EV effects on liver diseases. EVs purified from serum, stem cells, and hepatocytes were investigated in different experimental in vivo models of liver injury and in particular of liver fibrosis. Despite the different EV origin and the different types of injury (toxic, ischemic, diet induced, and so on), EVs showed an anti-fibrotic effect. In particular, EVs had the capacities to inhibit activation of hepatic stellate cells, one of the major players of liver fibrosis development; to reduce inflammation and apoptosis; to counteract the oxidative stress; and to increase hepatocyte proliferation, contributing to reducing fibrosis and ameliorating liver function and morphology.

## 1. Introduction

Different types of injury (ischemic, toxic, oxidative stress, and so on) may induce acute liver damage, which can elicit tissue inflammation. Inflammation, in turn, may contribute to further injury. When the injury or the inflammation persists, liver fibrosis may develop, eventually leading to end-stage liver disease, cirrhosis, and hepatocellular carcinoma. Although liver transplantation is the standard therapy for end-stage liver disease, its use is limited by insufficient availability of organs as well as by medical (immunosuppressive therapies) and financial considerations. To establish new anti-fibrotic and anti-inflammatory therapeutic strategies is one of the major clinical options to avoid transplantation. Preclinical studies have suggested that stem cells and their bio-products, in particular stem cell-derived extracellular vesicles (EVs), may represent a therapeutic option to treat or to alleviate liver fibrosis and inflammation.

## 2. Classification and Biogenesis of EVs

The term “EVs” was proposed in 2011 [1] to indicate vesicles enclosed by a lipid bilayer membrane and secreted into the extracellular space by virtually all cells in both physiological and pathological conditions. Therefore, EVs can be found in all biological fluids.

**Figure 1:**
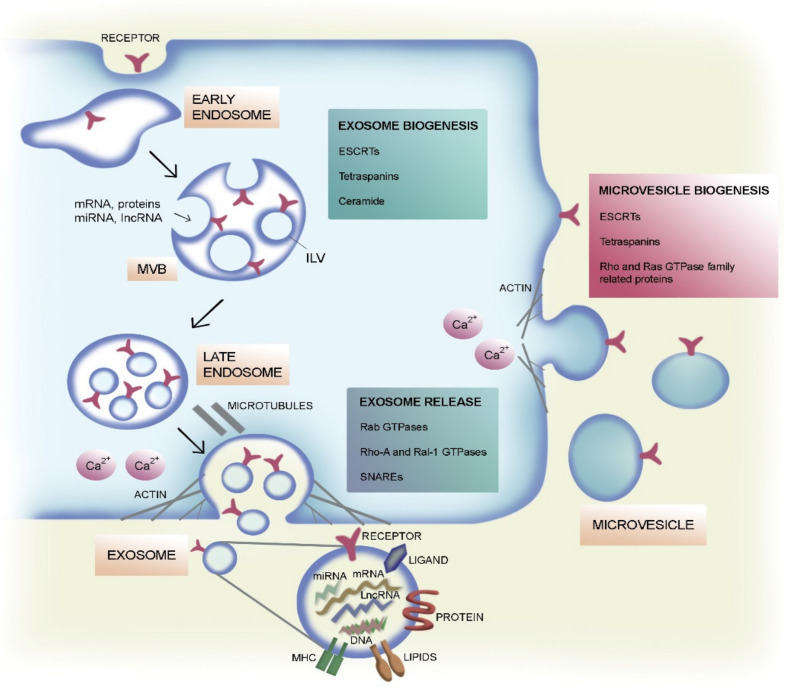
Extracellular vesicle (EV) biogenesis and secretion. Schematic representation of the formation and release of exosomes and microvesicles (MVs) by eukaryotic cells. Exosomes arise from intraluminal vesicles (ILVs) by budding into early endosomes and multivesicular bodies (MVBs). Several molecules are involved in ILV formation, in particular, lipids (e.g., ceramide) and proteins like tetraspanins (e.g., CD63, CD81, and CD9) and the endosomal sorting complex required for transport (ESCRT) machinery with its associated factors hepatocyte growth factor-regulated tyrosine kinase substrate (HRS), tumor susceptibility gene 101 protein (TSG101), apoptosis-linked gene-2 interacting protein X (ALIX), and vacuolar protein sorting-associated protein 4 (VPS4). Several Rab small guanosine triphosphatases (GTPases) (e.g., Rab11, Rab27a/b, and Rab35) contribute to transportation of MVBs to the plasma membrane, thus eliciting the exosome secretion. Other proteins involved in MVB fusion with the plasma membrane are Rho-A, Ral-1, and soluble N-ethylmaleimide-sensitive factor attachment protein receptors (SNAREs). Many of these proteins also participate in MV biogenesis, such as TSG101 through the interaction with arrestin domain-containing protein-1 (ARRDC1). Proteins associated to the Rho (ROCK) and the Ras GTPase family (ARF1, ARF6) also contribute to MV generation by controlling cytoskeleton rearrangements. Furthermore, a rise in calcium intracellular levels contributes to EV secretion. The molecular content of exosomes is also represented.

To date, the classification of EVs depends on their biogenesis, molecular composition, size, and density, and includes three main categories: exosomes, microvesicles, and apoptotic bodies [2,3]. Exosomes are small vesicles with an estimated size that ranges between 30 and 120 nm in diameter. They derive from intracellular trafficking through the endolysosomal pathway and their release is controlled by several cellular processes, including ceramide synthesis and calcium signaling [4]. Microvesicles (MVs), also known as microparticles or ectosomes, arise directly from the plasma membrane and range usually from 100 to 1000 nm in diameter. Whereas pre-apoptotic EVs have a larger size, those released from perfectly normal cells have a nano range size that may prevent size-dependent distinction from exosomes and are preferentially called ectosomes [5]. Lastly, apoptotic bodies are vesicles with a diameter of 1000–5000 nm. Apoptotic bodies arise from plasma membrane blebbing during apoptosis and represent fragments of dead cells [6]. These three EV categories are extremely heterogeneous in size and molecular content. Moreover, EVs isolated on the basis of their size have been shown to express several common protein markers [7] and a specific marker to distinguish one type of vesicle from another is still lacking. Finally, EV heterogeneity is increased by the lack of standardization in the isolation procedures, which include differential ultracentrifugation, density gradient separation, immunoaffinity purification, and size-exclusion chromatography [8]. Exosomes’ biogenesis begins with the generation of intraluminal vesicles (ILVs) from the inward and reverse membrane budding of intracellular endosomal structures, known as multivesicular bodies (MVBs). After fusion of MVBs with the cell surface, the ILVs contained within their lumen are released into the extracellular space, generating the exosomes [9] (Figure 1).

The endosomal sorting complex required for transport (ESCRT) machinery contributes to ILVs’ generation by controlling the selection of cargo (ESCRT-0), the intraluminal membrane budding (ESCRT-I and –II), and the membrane fission (ESCRT-III) [10,11,12]. The ESCRT complexes act in coordination with associated proteins required for exosome formation and secretion, such as hepatocyte growth factor-regulated tyrosine kinase substrate (HRS) [13,14], tumor susceptibility gene 101 protein (TSG101) [14], apoptosis-linked gene-2 interacting protein X (ALIX) [15], and vacuolar protein sorting-associated protein 4 (VPS4) [16]. However, exosome biogenesis can also occur in the absence of the ESCRT protein machinery [17]. An important role in exosome formation is played by tetraspanins (e.g., CD63, CD81, and CD9), transmembrane proteins particularly enriched in EVs [18,19,20,21]. Tetraspanins are mainly implicated in specific cargo sorting into ILVs [21,22,23,24] and in exosome release [25]. A lipid-driven mechanism has been also demonstrated [26]. In particular, exosome biogenesis may depend on ceramide synthesis, as the inhibition of neutral sphingomyelinase 2, an enzyme that converts sphingomyelin into ceramide, has proved to reduce exosome release [27,28]. Exosome secretion can be controlled by calcium. Treatment with calcium ionophores results in enhanced intracellular calcium levels, which prompt the exosome secretion [29,30]. Rearrangements in the actin and microtubule cytoskeleton also contribute to the transport of MVBs to the plasma membrane [31,32,33]. Among all proteins involved in exosome secretion, small guanosine triphosphatase (GTPase) RhoA [34], Ral-1 [35], and soluble N-ethylmaleimide-sensitive factor attachment protein receptors (SNAREs) [36,37] are required for MVB fusion with the plasma membrane. A growing body of research has focused on Rab GTPases involved in regulation of EV budding, transport along the cytoskeleton, and membrane fusion [38,39]. Examples of Rab GTPases associated with the exosome release are Rab11 [40], Rab27a [31,41,42], Rab27b [41], and Rab35 [43]. Despite being a different process from exosome formation, MV generation still involves the endosomal machinery, including several components of the ESCRT system, such as TSG101 and VPS4 [44]. Tetraspanins are also found in MVs, though less is known about their role in MV biogenesis [20]. MVs originate from the direct outward budding of the cell membrane [45] (Figure 1). In this process, several molecular modifications within the plasma membrane may occur, including changes in protein composition, rearrangements in lipid asymmetry and components, and a rise in calcium levels [45,46]. It has been demonstrated that the interaction between TSG101 and the arrestin domain-containing protein-1 (ARRDC1) causes the shift in TSG101 localization from endosomal membranes to the plasma membrane, thus resulting in membrane bending [45]. Moreover, the exposition of phosphatidylserine from the inner leaflet to the cell surface requires calcium-dependent enzymatic machineries, such as aminophospholipid translocases (flippases and floppases), scramblases, and calpain, which all contribute to the membrane bending process [47]. Like the exosome generation, MV biogenesis also requires the contribution of cytoskeletal proteins and their regulators. In particular, the Rho family of small GTPases and the Rho-associated protein kinase (ROCK) participate in MV generation by regulating actin dynamics [48]. A key regulator of MV shedding is the Ras-related GTPase ADP-ribosylation factor 6 (ARF6) [49]. Together with ARF1, ARF6 is involved in actin remodeling, cell invasion, and endocytic trafficking [50,51]. Their activation leads to the phosphorylation of myosin light chain and actomyosin contraction, thus resulting in the fission of MVs from the cell membrane [49,52].

In the last decades, EVs have emerged as important mediators of an evolutionary well-preserved mechanism of intercellular communication [53]. Following their release into the microenvironment, EVs, once internalized by endocytosis in target cell, can deliver selective patterns of proteins, bio-active lipids, and nucleic acids [54]. The mechanisms through which EVs interact with the cell surface are complex and mostly depend on the EV origin and recipient cells [55]. Proteins involved in EV–cell interaction include heparan sulphate proteoglycans [56,57], tetraspanins [58], lipids [59,60], and extracellular matrix receptors such as integrins [61,62,63]. EVs’ internalization within recipient cells may lead to direct cell stimulation, as well as transfer of receptors and biologically active molecules, such as cytosolic proteins, receptors, bioactive lipids, and nucleic acids [64,65,66]. Several studies have shown that EVs are particularly enriched in different RNA species, in particular mRNAs and microRNAs (miRNAs), that, when transferred to target cells, remained functional and can modify cellular behavior [65,67,68,69,70,71]. The EV encapsulation efficiently protects RNAs from the degrading activity of enzymes, like RNases, which are present in the extracellular space and in biological fluids [65,72]. It has been demonstrated that the RNA associated with EVs plays a pivotal role in mediating the therapeutic effects of EVs in different pathological conditions, including liver diseases and hepatic fibrosis. In particular, the EV-mediated transfer of non-coding RNAs, such as miRNAs, from stem cells and serum to the injured liver tissue has proved to be effective in ameliorating liver damage and fibrosis [73].

## 3. EVs to Treat Liver Diseases

**Figure 2:**
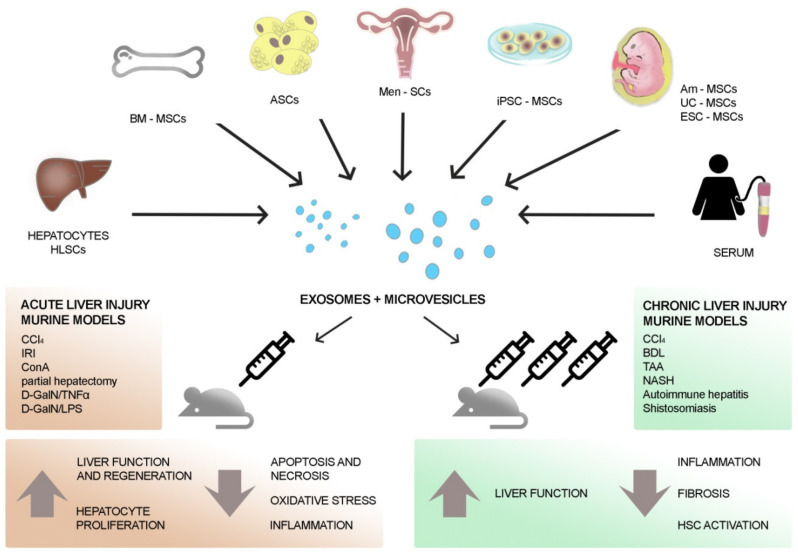
Therapeutic effects of EVs on hepatic injury. EVs secreted by mesenchymal stromal cells (MSCs) of different origin (such as liver, bone marrow, adipose tissue, menstrual blood, amnion, and umbilical cord) show therapeutic benefits on both acute and chronic liver injury models. Moreover, MSC-EVs derived from other stem cells (e.g., embryonic stem cell (ESC), induced pluripotent stem cells (iPSCs)), differentiated cells (e.g., hepatocytes), and serum contribute to the recovery of liver cells after damage. Acute liver injury models often foresee a single injection of EVs that prompts liver regeneration by supporting the proliferation of surviving liver cells and the reduction of oxidative stress and inflammation. Chronic liver injury models require several injections of EVs that restore liver function mainly through the reduction of hepatic fibrosis. CCl, carbon tetrachloride; IRI, ischemia-reperfusion injury; LPS, lipopolysaccharide; TNF, tumor necrosis factor; D-GalN, D-galactosamine; HLSC, human liver stem cell; hCB, human cord blood; hASC, human adipose stem cell; NASH, non-alcoholic steato-hepatitis; TAA, thioacetamide; BDL, bile duct ligation.

EVs of different origins contribute to the amelioration of acute liver injuries in different models of damage by improving hepatic oxidant injury, modulating the inflammatory response, and favoring hepatic cell proliferation and survival (Table 1 and Figure 2).

The first evidence that EVs can promote liver regeneration was shown by Herrera by injecting EVs derived from human liver stem cells (HLSCs), a mesenchymal stromal cell (MSC)-like population resident in human adult liver, in the model of 70% hepatectomy in rats. EVs accelerated liver functional and morphological recovery and increased hepatocyte proliferation. The beneficial effects were abrogated by EV pre-treatment with RNase, indicating a pivotal role of EV-associated RNAs in the pro-regenerative effect of EV treatment [74].

The administration of EVs derived from embryonic stem cell-derived MSCs (ESC-MSCs) was found to reverse carbon tetrachloride (CCl4) toxic-liver damage by increasing proliferation of the hepatocytes. The pro-proliferative effect of MSC-EVs was confirmed in different in vitro models of hepatocyte-injuries (acetaminophen and H2O2), where EV treatment up-regulated priming-phase genes, such as tumor necrosis factor alpha (TNF-alpha), interleukin 6 (IL-6), inducible nitric oxide synthase, cyclooxygenase 2, macrophage inflammatory protein 2, and anti-apoptotic proteins (e.g., Bcl-xL), favoring proliferation of survived hepatocytes [75]. Other sources of EVs have been tested in the CCl4 model. EVs fractionated by differential centrifugation from secretome of rat bone marrow (BM)-MSC were shown to attenuate liver injury, by partially restoring liver function and reducing oxidative stress in liver cells [87]. Treatment with EVs derived from human umbilical cord MSCs (hUC-MSC-EVs), either by tail vein administration or by oral gavage, rescued liver failure induced by CCl4, exerting anti-apoptotic and anti-oxidative effects. In particular, it has been demonstrated that the anti-oxidant effect was mediated by the delivery of glutathione peroxidase 1 (GPX1) protein, which reduced hepatic reactive oxygen species (ROS) and inhibited apoptosis induced by oxidative stress, via up-regulation of Bcl-2 and down-regulation of caspase 3 and 9 pathways. Down-regulation of GPX1 in hUC-MSCs and in derived EVs reverted the anti-apoptotic and anti-oxidant effects of hUC-MSC-EVs [82]. Moreover, in a murine model of CCl4, it has also been shown that treatment with hUC-MSC-EVs suppressed hepatic tumor development, through the anti-oxidant effect [77].

The effect of murine BM-MSC-EVs was also evaluated in a model of hepatic ischemia-reperfusion injury (IRI), where EVs were i.v. administrated 30 min prior to the surgical procedure. EV administration improved liver function and morphology by reducing the expression of inflammatory mediators both in vitro and in vivo. [78]. Compared with fibroblast-derived EVs, EVs obtained from BM-MSCs showed a stronger potential to attenuate liver injury and to improve organ regeneration after hepatic IRI [82]. Moreover, EVs obtained by hUC-MSCs can protect against hepatic IRI. In this case, EVs were administrated immediately after reperfusion and functional and histological improvements were observed 24 h after treatment. hUC-MSC-EVs induced a reduction in livers of neutrophil infiltrates; a decrease in mRNA expression level of IL-1-beta, IL-6, TNF-alpha, interferon (IFN)-gamma, and toll-like receptor 4 (TLR4); and alleviated oxidative stress. The authors found that hUC-MSC-EVs shuttled manganese superoxide dismutase (MnSOD), an anti-oxidant enzyme mainly located in the mitochondria. Of interest, knockdown of MnSOD in hUC-MSCs reduced in vivo anti-apoptotic and anti-oxidant effects of hUC-MSC-EVs, indicating a prominent role of this enzyme in the beneficial effect of EVs [81]. Moreover, EVs collected from induced pluripotent stem cells-derived MSCs (iPSC-MSCs) have proved to attenuate IRI [79,80]. The in vivo administration of iPSC-MSC-EVs in an IRI rat model reduced the loss of hepatocytes by necrosis and apoptosis, the infiltration of inflammatory cells, and the release of TNF-alpha and IL-6 inflammatory cytokines. Consistently, an increase in hepatocyte proliferation and an amelioration of hepatic damage by oxidative stress were observed after EV treatment [79].

EVs derived from stem cells were also tested in lethal models of hepatic injury (Table 1). BM-MSC-EVs effects were examined in a lethal murine model of hepatic liver failure induced by administration of D-galactosamine (D-GalN) and TNF-alpha. In untreated or vehicle-treated control groups, 24 h after damage, the survival was 0%. When EVs were administered immediately after injury, there was evidence of an improvement in the survival rate to 57% using murine (freshly or cryopreserved) BM-MSC-EVs and to 37.5% when human BM-MSC-EV were used. Improvement of survival was similar with either i.v. or i.p. administration. EV treatment reduced hepatic injury and modulated cytokines expression at the histological level [83]. Vesicles obtained from human menstrual blood-stem cells (MenSC-EV) showed a protective effect in fulminant hepatic failure induced by D-GalN and lipopolysaccharide (LPS). When EVs were administered 24 h before injury, the survival rate increased to 40%. MenSC-EVs ameliorated liver function, and reduced centrilobular focal necrosis, apoptosis, and inflammation. Moreover, EV treatment down-regulated hepatic and serum levels of pro-inflammatory cytokines (TNF-alpha, IL-6, -8 and 1-beta) [84]. Further, EVs obtained by human adipose stem cells (ASCs) rescued rats with D-GalN and D-GalN/LPS induced acute liver failure, promoting cell proliferation and exerting anti-inflammatory effect. Long-chain non coding (lncRNA) H19 shuttled by EVs was found to be involved in the pro-regenerative effect of ASC-EVs. In fact, EVs obtained from silenced ASCs for lncRNA H19 did not improve survival [85]. The immune-suppressive effect of EVs was also demonstrated on a murine hepatic injury model induced by concanavalin A (con-A), a lectin derived from jack beans. This particular type of liver injury is mediated by the activation of the adaptive immune system and the recruitment of T cells to the liver. In this context, three EV administrations of murine BM-MSC resulted in reduced liver necrosis, apoptosis, and inflammation, with an increased number of regulatory T cells among liver non-parenchymal cells. Moreover, EV treatment enhanced mRNA expression of the anti-inflammatory cytokines transforming growth factor (TGF)-beta and hepatocyte growth factor (HGF) [86].

EVs derived from murine hepatocytes, but not EVs derived from other terminal differentiated hepatic cells (Kupffer cells or sinusoidal endothelial cells), were found to promote regeneration in liver IRI and in 70% hepatectomy in vivo models [88]. In both experimental models, EV treatment increased hepatocyte proliferation dose-dependently. Hepatocyte-derived EVs shuttled to target hepatocyte specific sphingolipids and sphingolipid enzymes (neutral ceramidase and sphingosine kinase 2), inducing proliferation via synthesis of sphingosine-1-phosphatase expression [88]. A relevant role of sphingosine kinases and of phosphatase in hepatic regeneration induced by EVs was also confirmed using EVs derived from human iPSC-MSCs in an IRI model [80].

## 4. EVs to Treat Liver Fibrosis

The effects of EVs derived from different sources were also evaluated in different pre-clinical models of chronic liver injuries. EVs were found to counteract fibrosis by reducing hepatic inflammation and collagen deposition (Table 2 and Figure 2).

CCl4 is the most widely used hepatotoxin to develop liver fibrosis in different strains of mice and rats. EVs derived from different types of stem cells, but also from serum and from differentiated cells, were tested in this model. Firstly, EVs derived from hUC-MSCs were shown to be able to alleviate hepatic inflammation and collagen deposition in CCl4-induced fibrosis. At molecular level, collagen I and III and TGF-beta transcripts expression levels were reduced by EV-administration. Moreover, Smad2 phosphorylation, an important component of epithelial-to-mesenchymal transition (EMT)-pathway, was found to be reduced after EV-treatment [89]. EVs obtained by murine ASCs over-expressing miR-181-5p attenuated liver injury and down-regulated fibrotic transcripts, such as collagen I, vimentin, alpha-smooth muscle actin (alpha-SMA), and fibronectin, in CCl4-induced liver fibrosis. In vitro, miR-181-5p down-regulated signal transducer and activator of transcription (STAT)-3 and Bcl-2, thus suppressing hepatic stellate cells’ (HSCs) activation, and induced autophagy through the up-regulation of Beclin-1 [90]. Moreover, the lentiviral-driven miR-122 expression in ASCs enhanced their therapeutic effect in CCl4-induced liver fibrosis, by reducing HSC activation and collagen deposition. In vitro, ASC-EVs carrying miR-122 down-regulated the expression of several genes involved in proliferation and collagen maturation, such as cyclin G1 (CCNG1), insulin-like growth factor receptor 1 (IGF1R), and prolyl-4-hydroxylase a1 (P4HA1) [102]. EVs from murine iPSCs modulated in vitro HSC activation and had an anti-fibrotic effect not only in CCl4 murine model, but also in bile duct ligation (BDL)-induced liver fibrosis. Molecular analyses of liver tissues demonstrated down-regulation of profibrogenic genes alpha-SMA, collagen I, and tissue inhibitor of metalloproteinase 1 (TIMP-1) [91]. In CCl4-induced chronic liver disease, EVs from amnion-derived MSCs (Am-MSC) reduced fibrosis, Kupffer cell number, and HSC activation. The in vitro effects of EVs on Kupffer cells were further investigated. Am-MSC-EV treatment reduced the expression level of pro-inflammatory molecules, such as TNF-alpha, IL-1-beta, and MCP-1, in Kupffer cells stimulated with LPS. The increase in nuclear factor kappa-light-chain-enhancer of activated B cells (NF-kB) transcriptional activity induced by LPS was reduced by Am-MSC-EV treatment, through the inhibition of the phosphorylation of IkB-alpha and p65. As Am-MSC-EV did not decrease NF-kB transcriptional activity induced by TNF receptor associated factor (TRAF), the authors suggested that AMSC-EV may suppress the earlier steps of the LPS/TLR4 signaling pathway [92]. EVs derived from human BM-MSCs have shown similar effects in a rat model of CCl4 -induced liver fibrosis, and the reduced HSC activation has been linked to the inhibition of several genes in the Wnt signalin pathway, such as peroxisome proliferator-activated receptor (PPAR)-gamma, beta-catenin, WNT3a, and WNT10b. Moreover, the EV-treated animals exhibited reduced mRNA expression levels of inflammatory cytokines IL-1, IL-2, IL-6, IL-8, IL-10, and TNF-alpha [93]. Serum EVs from healthy subjects dose-dependently inhibited fibrosis in CCl4-injured liver and reduced alpha-SMA expression, indicating a reduction of HSC activation. Protein array showed a reduction of some pro-inflammatory cytokines and chemokines, such as IFN-gamma, IL-2, IL-4, and TNF-alpha in liver tissue, as in the circulation of CCl4-treated mice. Different miRNAs shuttled by serum EVs have a predicted anti-fibrotic effect. Among them, miR-34c-3p, -151-3p, -483-5p, -532-5p, and -687 have been demonstrated to be implicated in the anti-fibrotic effect of serum-EVs using specific miRNA-mimics in in vitro experiments on activated HSCs [94]. Moreover, pro-fibrotic genes alpha-SMA, connective tissue growth factor (CCN2), and collagen I induced by CCl4 were dose-dependently reduced by treatment with hepatocyte EVs. Moreover, inflammatory response to CCl4 was suppressed by hepatocyte EV treatment. In particular, a reduction of the frequency of hepatic monocytes and macrophages was reduced by hepatocyte EV treatment. Protein microarray analysis indicated that hepatocyte EV administration reduced levels of the fibrosis-related factors TIMP-1, chemokine (C-C motif) ligand (CCL)-3, CCL-5, and CCL-12. Interestingly, RNA sequencing of liver tissues revealed that hepatocyte-EV treatment induced significant differences in the expression of 233 CCl4-regulated genes. These were associated with fibrosis, cell division, extracellular matrix, drug detoxification, membrane trafficking, and immunity [95].

In a thioacetamide (TAA)-induced chronic liver injury model, an anti-fibrotic effect of EVs obtained from hESC-derived MSCs has been reported. Molecular analyses showed up-regulation of matrix metalloproteinase (MMP) 9 and 13, anti-apoptotic genes, and anti-inflammatory cytokines (IL-10 and TGF-beta) with concomitant down-regulation of collagen, alpha-SMA, and TIMP-1 transcripts, and of pro-apoptotic and pro-inflammatory genes (TNF-alpha and IL-2) [96]. Rapid clearance of EVs from target organ may reduce the efficiency of EV treatments. For this reason, in a TAA model of hepatic fibrosis, the possibility of using EVs encapsulated in polyethylene glycol macromeres (gel-EVs) was tested. In this condition, the gel-EVs were swollen gradually and the EVs were progressively released over 1 month and accumulated in liver, as indicated by in vivo tracking experiments. Histological and molecular analyses demonstrated superior anti-fibrotic, anti-apoptotic, and anti-inflammatory effects of gel-EVs in comparison with free-EVs [97]. EVs derived from human cord perivascular cells (HUCPVCs), an alternative source of MSCs, were also tested in a TAA-chronic liver injury model. HUCPVC-EVs over-expressing insulin growth factor 1 (IGF-1) showed, in comparison with naive HUCPVC-EVs, a stronger anti-fibrotic effect and were the only ones able to reduce the activation of HSCs [98].

Different types of EVs have been also tested in different animal models of non-alcoholic steatohepatitis (NASH). Am-MSC-EVs improved histological findings and pro-inflammatory factors expression in rats with NASH induced by feeding them with high-fat diet for four weeks. Am-MSC-EVs suppressed the activation of pro-inflammatory M1 macrophages and down-regulated the expression of pro-inflammatory cytokines such as TNF-alpha, IL-1-beta, and IL-6 [92]. EVs derived from HLSCs influenced the progression of NASH induced by a diet deprived of methionine and choline. HLSC-EVs treatment improved liver function and morphology by reducing liver fibrosis and inflammation. At molecular level, 28 out of 29 fibrosis-associated genes up-regulated in NASH livers were significantly down-regulated by HLSC-EV administration. The list of reverted genes included collagen 1, alpha-SMA, TGF-beta, and the gene latent-transforming growth factor beta-binding protein 1 (Ltbp1), genes involved in tissue remodeling (TIMP-1 and MMP-1a, -13, -14, and -8) and in inflammation (TNF-alpha and IL-1-beta). The anti-inflammatory effect of HLSC-EV-treatment was also indicated by the reduction of inflammatory cells accumulated in the liver and by the increase in IL-10 expression level in NASH mice treated with HLSC-EVs [100].

Chronic inflammation due to autoimmune hepatitis may cause development of liver fibrosis. In a murine model of autoimmune hepatitis, induced by intraperitoneal injection of S100 liver antigen, EVs derived from naive BM-MSC or from BM-MSC transfected with miR-223 were able to revert liver injury by regulation of inflammasome NLRP3 and caspase-1 [99]. Schistosomiasis is another cause of chronic liver inflammation. Recently, EVs obtained from hUC-MSC have proved to alleviate S. japonicum-induced liver injury, thus increasing the survival of schistosome-infected mice. In vivo administration of hUCMSC-EV reduced hepatic fibrosis by down-regulating the expression of alpha-SMA, collagen I, and collagen III. Moreover, a decrease in mRNA expression levels of IFN-gamma, TNF-alpha, and IL-beta has also been observed after hUCMSC-EV administration [101].

## 5. Advantages and Need for Future Clinical Applications of EVs

Compared with cell-based therapy, EVs therapeutic approach has some advantages. EVs exhibit a superior efficacy profile of cell-based therapy as they pass biological barriers and act as effective carriers of different molecules (RNAs, proteins, and lipids). EVs appear more stable and suitable for long term storage, in comparison with the cells of origin. Interestingly, the possibility to lyophilize purified EVs has recently been shown [103]. This could allow the production of ready-to-use batches of EVs that can be easily transported. However, further studies are needed to prove that lyophilization does not alter the EVs’ morphology and functionality. Moreover, no adverse immune responses have been reported in patients undergoing allogeneic administration of MSC-EVs [104] and a significant benefit of MSC-EVs in respect to MSC-treatment is the chance to avoid potential tumorigenicity of the cells of origin. Actually, no evidence of oncogenic potential of MSC-EVs has been reported and in vitro and in vivo experiments indicated that MSC-EVs can inhibit tumor growth by interfering with cell cycle and inducing apoptosis and/or necrosis of different cancer cell lines [105]. Anti-tumor activity has also been reported for HLSC-EVs [106].

A lot of challenges need to be addressed prior to the application of EVs in clinical trials. First, the choice of the best EV source must be established. EVs can be obtained from different cellular sources, in particular from various type of stem cells maintained in different culture conditions (e.g., hypoxia, growth factors), which may modify the contents of released EVs and influence their effects in tissue regeneration [107]. Despite these differences, all these studies indicated that EVs may be a promising cell-free treatment for liver diseases. Even though a direct comparison of the different cell sources is still missing, we can speculate that hUC-MSCs could be one of the best cellular sources, being more accessible in respect to BM-MSCs and not involving ethical objections, as reported for ESCs and iPSCs. Moreover, HLSCs are a good candidate stem cell source to obtain EVs for tissue regeneration. HLSC-EVs have been tested in different experimental animal models of acute and chronic liver disease [74,100]. Of interest, HLSC-EVs have also been used in chronic kidney diseases, such as diabetic nephropathy and aristolochic acid-induced nephropathy, both characterized by fibrosis development. In these two animal models, HLSC-EVs exhibited similar therapeutic effects of BM-MSC-EVs, despite the different molecular mechanisms [108,109,110].

Moreover, the best way to obtain the quantity of EVs necessary for clinical application has to be standardized. In addition, it is necessary to validate the dosage and the half-life of EVs, freshly isolated or after cryopreservation or lyophilization. The content of EVs needs to be deeply investigated, in order to understand which EV components could act as pro-regenerative or anti-fibrotic factors and which could possibly be harmful. Moreover, the unknown negative effects of single or repeated administration have to be clarified.

## 6. Conclusions 

EVs from various sources, such as serum, hepatic cells, and different type of stem cells (embryonic, adult, bone marrow, liver), have been tested in different animal models of liver disease using different doses, regimens, and time of administration. EVs of different origins appear to attenuate fibrosis in pre-clinical models mimicking different pathogenic conditions. EVs contain biologically active molecules such as proteins, mRNAs, and non coding RNAs that can play critical roles in modulating immune cells, leading to reduced inflammation and consequently fibrosis. EVs can also directly modify the activation state of the HSCs, reducing the deposition of fibrotic factors and secretion of pro-inflammatory molecules.

**Figure 3:**
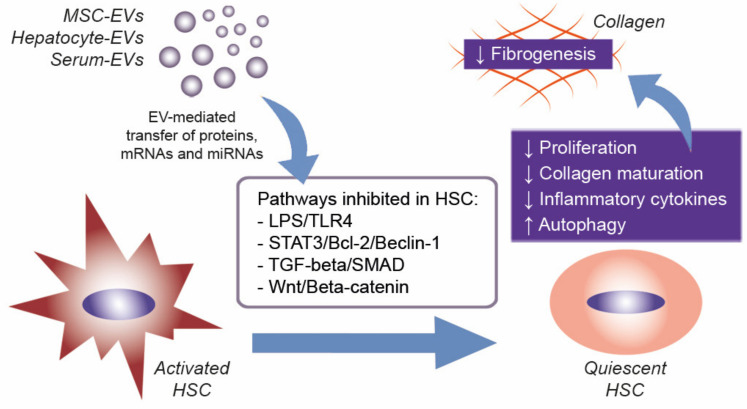
EV effects on HSC during liver fibrosis. EVs derived from different sources, such as MSCs, hepatocytes, and serum, contain proteins and specific patterns of mRNAs and miRNAs that can modulate a number of molecular pathways in target cells. In liver fibrosis, the possible EV uptake by activated HSC could revert its quiescent state, thus reducing HSC activation and fibrogenesis. TGF, transforming growth factor; TLR4, toll-like receptor 4; SMAD, small mothers against decapentaplegic; STAT, signal transducer and activator of transcription.

Despite that the benefits of EVs in different models of liver damage, in particular in fibrosis, have been extensively evaluated, further studies are needed to elucidate the mechanisms of action. One possible target of therapeutic EVs in liver fibrosis could be the HSC. The activation of HSC is a critical event during liver fibrosis and is controlled by several cytokines, growth factors, and small RNA species such as miRNAs [73]. The molecular content of EVs derived from MSCs, hepatocytes, and serum may suppress the myofibroblast-like phenotype of HSC through the inhibition of different molecular pathways mainly involved in HSC proliferation and collagen synthesis (Figure 3). In liver fibrosis, the therapeutic effect of EVs could be attributed, at least in part, to the action of EV-associated miRNAs that are down-regulated in HSC during fibrosis [90,94,99,102]. One could speculate that EVs could alleviate hepatic fibrosis by restoring physiological expression levels of miRNAs in liver tissue.

The preclinical data summarized in this review support the idea that cell-free therapy, with different types of EVs, could be a novel alternative therapeutic approach for liver diseases and in particular for liver fibrosis. However, before implementing this promising approach, numerous problems have to be solved, such as large-scale EV production and safety.

## Figures and Tables

**Table 1:** Effects of extracellular vesicles (EVs) of different origins on different models of acute liver injuries. CCl4, carbon tetrachloride; IRI, ischemia-reperfusion injury; LPS, lipopolysaccharide; TNF, tumor necrosis factor; D-GalN, D-galactosamine; HLSC, human liver stem cell; MSC, mesenchymal stromal cell; ESC, embryonic stem cell; hUC, human umbilical cord; hASC, human adipose stem cell.

In Vivo Models of Liver Disease	EV Sources	Route and Time of EV Administration	Effects of EV Administration	References
Partial hepatectomy	HLSCs	Tail vein immediately after injury	Pro-proliferative and anti-apoptotic effect on hepatocytes	[74]
CCl_4_	ESC-MSCs	Intra-splenic injection simultaneously with the damage	Pro-proliferative effect on hepatocytes	[75]
CCl_4_	hUC-MSCs	Tail vein or oral gavage 24 h post-CCl_4_	Anti-oxidant and anti-apoptotic effects on hepatocytes	[76]
CCl_4_	hUC-MSCs	Tail vein 24 h post-CCl_4_	Inhibition of inflammation, oxidative stress, and apoptosis. Suppression of hepatic tumor development	[77]
IRI	Murine BM-MSCs	Tail vein 30 min before surgery	Reduction of liver necrosis and hepatocyte apoptosis by modulating inflammation. Improvement of liver function	[78]
IRI	hiPSC-MSCs	Inferior vena cava immediately after reperfusion	Reduce histological damage, inflammation, apoptosis, and oxidative stress, and improve hepatic function	[79,80]
IRI	hUC-MSCs	Tail vein immediately after surgery	Reduce apoptosis, neutrophilic infiltrates, and oxidative stress	[81]
IRI	Human BM-MSCs/ fibroblasts	Inferior vena cava before surgery	Reduction of liver necrosis and inflammation. Improvement of liver function and regeneration	[82]
D-GalN/TNF-alpha	Murine/human BM-MSCs	Tail veil or intraperitoneal injection immediately after damage	Increase in mice survival, reduction of hepatic inflammation and injury	[83]
D-GalN/LPS	Men-SCs	Tail vein 24 h before injury	Improvement of liver function and survival, inhibition of apoptosis	[84]
D-GalN/LPS	hASC	Iliac vein 24 h after injury	Reduction of necrosis and inflammation	[85]
ConA	Murine BM-MSCs	Intravenous EV injection of 20 μg/mL at 0, 8, and 16 h after injury	Reduction of hepatic necrosis, apoptosis, and inflammation	[86]
IRI and partial hepatectomy/ CCl_4_	Rat BM-MSCs	A single injection via hepatic portal vein of 500 μg/mL of exosome-rich fractionated secretome before removing the clamp (IRI) or 24 h post-CCl_4_	Improvement of hepatic regeneration and function, reduction of oxidative stress	[87]
IRI and partial hepatectomy	Murine hepatocytes	Intravenously EV injection 24 and 48 h after IRI, 24 h after hepatectomy	Increase of hepatocyte proliferation	[88]

**Table 2:** Effects of EVs of different origins on different models of fibrotic liver damages. NASH, non-alcoholic steato-hepatitis; TAA, thioacetamide; HUCPVC, human cord perivascular cell; IGF, insulin-like growth factor; EMT, epithelial-to-mesenchymal transition; SMA, smooth muscle actin.

In Vivo Model of Liver Fibrosis	EV Sources	Route and Time of EV- Administration	Effects of EV-Administration	References
CCl_4_	hUC-MSCs	Single EV-dose directly injected into left and right hepatic lobes, 6 weeks after CCl_4_ treatment	Inhibition of EMT and protection of hepatocytes	[89]
CCl_4_	miR-181-5p modified murine ASCs	Intrasplenic injection twice each week for 8 weeks concomitantly with CCl_4_ treatment	Anti-fibrotic effect, amelioration of liver function	[90]
CCl_4_ BDL	human-iPSCs	Tail vein three times a week for the last two weeks of the CCl_4_ study; tail vein daily injection for the last six days of duct ligation	Reduction of fibrosis and HSC activation	[91]
CCl_4_ NASH	hAm-MSCs	Intravenous injection at week 3 after the start of CCl_4_ treatment and at week 3 and 4 after starting the high fatty diet to induce NASH	Reduction of Kupffer cells, of expression levels of pro-inflammatory and pro-fibrotic cytokines, and of HSC activation	[92]
CCl_4_	Human BM-MSCs	Single EV injection through the tail vein 8 weeks after CCl_4_ treatment	Improvement of liver function and reduction of fibrosis, inflammation, and HSC activation via Wnt/beta-catenin pathway	[93]
CCl_4_ TAA	Murine and human serum	Intraperitoneal administration three times per week during the last two to three weeks of CCl_4_ treatment; intraperitoenal administration every day during the last week of thioacetic treatment experiment	Reduction of the levels of hepatocyte death, inflammatory infiltrates, AST and ALT, pro-inflammatory cytokines, and HSC	[94]
CCl_4_	Murine and human hepatocytes	Intraperitoneal administration three times per week during the last two weeks of the experiment	Reduction of alpha-SMA expression and of fibrosis and inflammation	[95]
TAA	hESC-MSCs	Intrasplenic injection	Reduction of fibrosis and immune cell infiltration, up-regulation of anti-apoptotic and anti-inflammatory genes	[96]
TAA	hESC-MSCs	Intraperitoneal injection of free or hydrogel-loaded EVs	Reduction of necrosis, inflammation, and fibrosis	[97]
TAA	HUCPVCs engineered to produce IGF-1	On week 6 of treatment, tail vein injection every 5 days (total of three doses)	Reduction of collagen deposition and expression of fibrogenic transcripts	[98]
Autoimmune hepatitis	Murine BM-MSCs engineered with miR-223	Administration of EVs at day 21, 28, and 35	Improvement of liver structure and function and of lymphocyte infiltration	[99]
NASH	HLSCs	Intravenous twice a weeks starting from week 2 of diet	Improvement of liver function and reduction of fibrosis and inflammation	[100]
Schistosomiasis	hUCMSCs	Intravenous injection at the fourth or at the sixth week after infection	Increased mice survival, improvement of liver function, and reduction of fibrosis and inflammation	[101]

## Abbreviations

EVs	Extracellular Vesicles
MVs	Microvesicles
ILVs	Intraluminal Vesicles
MVBs ESCRT	Multivesicular Bodies Endosomal Sorting Complex Required for Transport
HRS	Hepatocyte Growth Factor-Regulated Tyrosine Kinase Substrate
TSG101	Tumor Susceptibility Gene 101 Protein
ALIX	Apoptosis-Linked Gene-2 Interacting Protein X
VPS4	Vacuolar Protein Sorting-Associated Protein 4
GTPase	Guanosine Triphosphatase
SNAREs	Soluble N-Ethylmaleimide-Sensitive Factor Attachment Protein Receptors
ARRDC1	Arrestin Domain-Containing Protein-1
ROCK	Rho-Associated Protein Kinase
ARF	ADP-Ribosylation Factor
miRNA	MicroRNA
HLSCs	Human Liver Stem Cells
MSCs	Mesenchymal Stromal Cells
ESC-MSCs	Embryonic Stem Cell-Derived MSCs
CCl_4_	Carbon Tetrachloride
TNF-alpha	Tumor Necrosis Factor Alpha
IL	Interleukin
BM-MSCs	Bone Marrow MSCs
GPX1	Glutathione Peroxidase 1
ROS	Reactive Oxygen Species
IRI	Ischemia Reperfusion Injury
IFN	Interferon
MnSOD	Manganese Superoxide Dismutase
iPSC-MSCs	Induced Pluripotent Stem Cell-Derived MSCs
D-GalN	D-Galactosamine
Men-SCs	Menstrual Stem Cells
LPS	Lipopolysaccharide
ASCs	Adipose Stem Cells
lncRNA	Long Non-Coding RNA
conA	Concanavalin A
TGF	Transforming Growth Factor
HGF	Hepatocyte Growth Factor
TGF-beta	Trasforming Growth Factor Beta
AST	Aspartate Transaminase
ALT	Alanine Transaminase
EMT	Epithelial to Mesenchymal Transition
alpha-SMA	Alpha Smooth Muscle Actin
HSCs	Hepatic Stellate Cells
TIMP-1	Tissue Inhibitor of Metalloproteinase-1
CCNG1	Cyclin G1
IGF1R	Insulin-Like Growth Factor Receptor 1
P4HA1	Prolyl-4-Hydroxylase A1
Am-MSCs	Amnion-Derived MSCs
PPAR	Peroxisome Proliferator-Activated Receptor
CCN2	Connective Tissue Growth Factor
TAA	Thioacetamide
MMP	Matrix Metalloproteinase
gel-EVs	EVs Encapsulated in Polyethylene Glycol Macromeres
HUCPVCs	Human Umbilical Cord Perivascular Cells
IGF-1	Insulin Like Growth Factor 1
NASH	Non-Alcoholic Steato-Hepatitis
hUC-MSCs	Human Umbilical Cord MSCs
BDL	Bile Duct Ligation
TLR4	Toll-Like Receptor 4
STAT	Signa Transducer and Activation of Transcription
NF-kB	Nuclear Factor Kappa-light-chain-enhancer of Activated B Cells
TRAF	TNF Receptor Associated Factor
CCL	Chemokine (C-C motif) Ligand
SMAD	Small Mothers Against Decapentaplegic
